# Impact of AJCC prognostic staging on prognosis and postmastectomy radiotherapy decision-making in hormone receptor-positive and HER2-positive breast cancer

**DOI:** 10.1093/bjsopen/zrac025

**Published:** 2022-04-25

**Authors:** Guan-Qiao Li, Yang Yu, Wen-Wen Zhang, Ping Zhou, Chen-Lu Lian, Zhen-Yu He, San-Gang Wu

**Affiliations:** 1 Department of Breast Surgery, Hainan General Hospital (Hainan Affiliated Hospital of Hainan Medical University), Haikou, People’s Republic of China; 2 Department of Radiation Oncology, National Cancer Center/Cancer Hospital, Chinese Academy of Medical Sciences (CAMS) and Peking Union Medical College (PUMC), Beijing, People’s Republic of China; 3 Department of Radiation Oncology, The First Affiliated Hospital of Xiamen University, Xiamen, People’s Republic of China; 4 Department of Radiation Oncology, Sun Yat-sen University Cancer Center, State Key Laboratory of Oncology in South China, Collaborative Innovation Center of Cancer Medicine, Guangzhou, People’s Republic of China

## Abstract

**Background:**

The role of postmastectomy radiotherapy (PMRT) in patients with node-positive hormone receptor-positive (HoR) and HER2-positive breast cancer (BC) regarding AJCC pathological prognostic staging (PPS) has not been fully determined. This study aimed to validate PPS in patients with node-positive HoR^+^/HER2^+^ BC after mastectomy and to investigate the role of PPS on PMRT decision-making in this patient subset.

**Methods:**

Patients diagnosed with BC from the Surveillance, Epidemiology, and End Results database were included. Patients were classified based on the anatomical staging (AS) and PPS. Breast cancer-specific survival (BCSS) was calculated.

**Results:**

In total, 6862 patients were included: 4306 (62.8 per cent) patients received PMRT and 2556 (37.2 per cent) patients had not. Compared to AS, PPS downstaged 5260 patients (76.7 per cent) and no patients were upstaged. The C-index was similar between PPS and AS (0.690 *versus* 0.682; *P* = 0.346). Regarding AS, patients who received PMRT had significantly better BCSS than those who had not in stage IIIA (*P* = 0.017) and stage IIIC (*P* < 0.001) disease, but not in stage IB (*P* = 0.675), IIA (*P* = 0.677), IIB (*P* = 0.100), and IIIB (*P* = 0.747) disease. Regarding PPS, patients who received PMRT had significantly better BCSS than those who had not in stage IIIA (*P* = 0.038) and stage IIIB (*P* = 0.017) disease, but not in stage IA (*P* = 0.336), IB (*P* = 0.893), IIA (*P* = 0.815), and IIB (*P* = 0.120) disease. PPS might allow approximately 1390 stage III patients (45.0 per cent) in the AS criterion to avoid PMRT.

**Conclusion:**

PPS does not provide better risk discriminatory ability in predicting prognosis than AS in patients with node-positive HoR^+^/HER2^+^ BC after mastectomy. However, PPS is valuable in providing prognostic counselling to patients and may also guide PMRT decision-making.

## Teaser

AJCC pathological prognostic staging downstaged 74.7 per cent of patients with breast cancer with hormone receptor-positive and HER2-positive disease but does not provide better risk discriminatory ability in predicting prognosis than anatomical staging. However, pathological prognostic staging is valuable in providing prognostic counselling to patients and may also guide postmastectomy radiotherapy decision-making.

## Introduction

The traditional breast cancer (BC) AJCC staging was defined by anatomical stage (AS), which was based on information regarding tumour size (T), regional nodal metastasis (N), and distant metastasis (M)^1^. Although AS has been widely used in past decades, questions were raised about whether AS could accurately define the prognosis of BC in the modern era. With advances in BC biology, several biological markers have been identified and validated to describe prognosis and guide treatment decision-making^2–4^. The 8th edition of the AJCC BC pathological prognostic staging (PPS) was first introduced into clinical use in 2017, and incorporated contemporary biological factors, including tumour grade, human epidermal growth factor receptor-2 (HER2), oestrogen receptor (ER), and progesterone receptor (PR) status into the traditional AS system^5^. Several studies have confirmed that the new AJCC PPS allows for more refined risk stratification regarding survival for patients with BC receiving appropriate multimodal therapy^6–11^.

For oncologists, it is important to develop a staging system that provides information that precisely defines prognosis. A valuable staging system should also provide effective guidance for selection of appropriate treatment. Previous studies have found that the new PPS may have an important role in treatment decision-making for stage I to III BC^10,11^. However, a recent study showed that PPS did not provide better discriminatory ability of risk stratification compared with the AS in T1-2N0M0 triple-negative BC, but PPS could more accurately predict the efficacy of chemotherapy^12^. In those with hormone receptor-positive (HoR^+^) and HER2-positive (HER2^+^) disease, whether PPS exhibits a superior risk stratification than AS is still debatable^13,14^. The findings from the ShortHER trial also did not support PPS as guidance to de-escalate anti-HER2 treatment for HER2^+^ patients^14^.

HoR^+^/HER2^+^ BC is a clinical subtype with biological features and therapeutic responses that differ from other subtypes of BC. Enormous progress has been made in the understanding and treatment of HoR^+^/HER2^+^ BC in the last 30 years that have contributed to survival benefit for affected patients^15^. Advances in anti-HER2 treatment have significantly improved the survival outcomes for patients with HER2^+^ BC over time^15^. Postmastectomy radiotherapy (PMRT) is commonly used in conjunction with chemotherapy, hormone therapy, and anti-HER2 treatment, to decrease locoregional recurrence (LRR) and improve survival outcomes in node-positive HoR^+^/HER2^+^ BC^16^. However, the role of PMRT in this patient subset regarding PPS has not been fully determined. This study aimed to validate PPS in patients with node-positive HoR^+^/HER2^+^ BC after mastectomy and investigate the role of PPS in PMRT decision-making in this patient subset.

## Methods

### Data source and study population

Patients with BC diagnosed between 2010 and 2018 from the Surveillance, Epidemiology, and End Results (SEER) programme were included. SEER is a population-based database on cancer incidence and survival outcomes in the USA, which includes approximately 47.9 per cent of the US population^17^. The SEER Program routinely collects information regarding patient demographics, tumour location, stage at diagnosis, histology, the first course of treatment, and vital status.

Patients who met the following criteria were identified: women with node-positive BC; aged 18 to 69 years; treated with mastectomy and chemotherapy with or without PMRT; the pathological diagnosis was invasive ductal carcinoma (IDC), invasive lobular carcinoma (ILC), or mixed IDC and ILC; HoR^+^ (ER^+^ and PR^+^ or PR^–^) and HER2^+^ disease; and available data regarding T stage, N stage, grade, HER2, ER, and PR status. Patients with stage T0 disease, M1 stage disease, receiving preoperative radiotherapy, and receiving non-beam external irradiation were excluded. This study did not require institutional review board approval because the data were deidentified in the SEER Program.

### Variables and outcome

The following data were collected from the SEER Program: age, race, histology, T stage, N stage, grade, PR status, and receipt of PMRT. All patients were restaged retrospectively. PPS was determined based on the AJCC 8th edition Staging Manual, and the AS according to pathological T and N stage was consistent with the 7th edition Staging Manual^5^. The primary endpoint of this study was BC-specific survival (BCSS), defined as the time from the initial diagnosis of BC to the death from BC.

### Statistical analysis

A χ^2^ test was performed to determine the factors significantly related to PMRT compliance. The Harrell concordance index (C-index) and the Akaike Information Criterion (AIC) were calculated to compare the discriminatory ability for the two staging systems^18,19^. A higher C-index shows a better predictive value and a lower AIC demonstrates superior model fit. Survival data were analysed using Kaplan–Meier curves, and log-rank tests were conducted to compare the survival distributions. Cox proportional hazards regression was used to determine independent prognostic factors associated with BCSS. Sensitivity analyses after stratification of the AS and PPS systems were performed to further determine the specific subgroups benefiting from PMRT. All analyses were conducted using SPSS^®^ version 22 (IBM, Armonk, New York, USA), MedCalc Statistical Software version 18.2.1 (MedCalc Software, Ostend, Belgium), or R version 3.0 (R Foundation for Statistical Computing, Vienna, Austria). All *P* values < 0.05 were considered to be statistically significant.

## Results

### Patient baseline characteristics

**Fig. 1:**
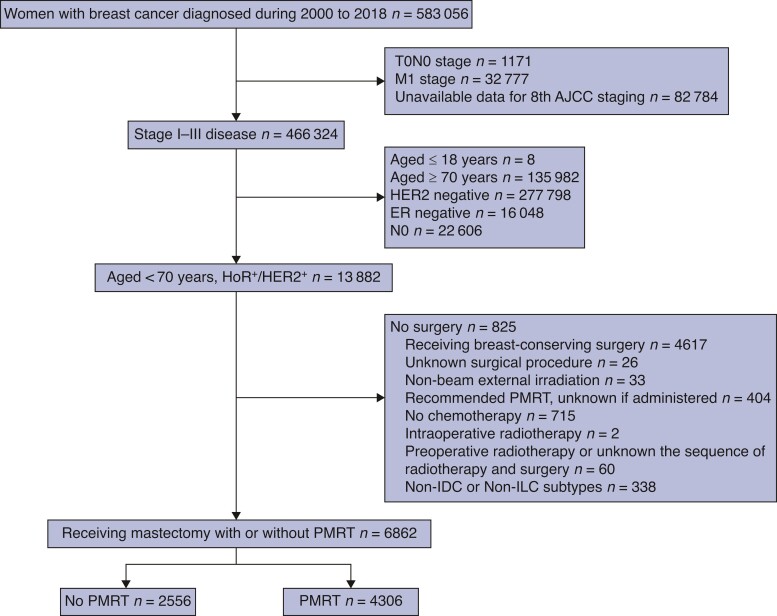
Flow diagram of the study cohort PMRT, postmastectomy radiation; IDC, invasive ductal carcinoma; ILC, invasive lobular carcinoma.

In total, 6862 patients were included in this study (*Fig. 1*). Patient baseline characteristics are listed in *Table 1*. Altogether, 6219 patients had IDC subtype (90.6 per cent), 4872 had stage N1 disease (71.0 per cent), and 3971 had poorly/undifferentiated tumours (57.9 per cent). Regarding HoR status, 1787 patients (26.0 per cent) had PR-negative disease. In total, 4306 (62.8 per cent) had received PMRT. Younger age (*P* < 0.001), advanced T stage (*P* < 0.001), advanced N stage (*P* < 0.001) and PR-positive disease (*P* = 0.010) were factors related to compliance with PMRT.

**Table 1:** Patients’ baseline characteristics

Variables	*n*	No PMRT (%)	PMRT (%)	*P*
**Age (years)**				
<50	3162	1050 (41.1)	2112 (49.0)	<0.001
≥50	3700	1506 (58.9)	2194 (51.0)	
**Race**				
Non-Hispanic white	4085	1510 (59.1)	2575 (59.8)	0.286
Non-Hispanic black	844	300 (11.7)	544 (12.6)	
Hispanic (all races)	1083	429 (16.8)	654 (15.2)	
Other	850	317 (12.4)	533 (12.4)	
**Histology**				
IDC	6219	2324 (90.9)	3895 (90.5)	0.112
ILC	283	90 (3.5)	193 (4.5)	
Mixed IDC and ILC	360	142 (5.6)	218 (5.1)	
**Grade**				
Well differentiated	217	82 (3.2)	135 (3.1)	0.469
Moderately differentiated	2674	973 (38.0)	1702 (39.5)	
Poorly/undifferentiated	3971	1502 (58.8)	2469 (57.3)	
**Tumour stage**				
T1	1701	853 (33.4)	848 (19.7)	<0.001
T2	3281	1250 (48.9)	2031 (47.2)	
T3	1246	315 (12.3)	931 (21.6)	
T4	634	138 (5.4)	496 (11.5)	
**Nodal stage**				
N1	4872	2106 (82.4)	2766 (64.2)	<0.001
N2	1279	286 (11.2)	993 (23.1)	
N3	711	164 (6.4)	547 (12.7)	
**PR status**				
Negative	1787	711 (27.8)	1076 (25.0)	0.010
Positive	5075	1845 (72.2)	3230 (75.0)	

PMRT, postmastectomy radiotherapy; IDC, invasive ductal carcinoma; ILC, invasive lobular carcinoma; T, tumour; N, nodal; PR, progesterone receptor.

### Staging migration

Using the 7th AJCC AS criterion, 300 (4.4 per cent), 1086 (15.8 per cent), 2383 (34.7 per cent), 1870 (27.3 per cent), 512 (7.5 per cent), and 711 (10.4 per cent) patients had stage IB, IIA, IIB, IIIA, IIIB, and IIIC disease, respectively. No patients had stage IA disease, using the AS criterion. Regarding the 8th AJCC PPS criterion, 1169 (17.0 per cent), 2437 (35.5 per cent), 959 (14.0 per cent), 594 (8.7 per cent), 824 (12.0 per cent), and 879 (12.8 per cent) patients were staged as IA, IB, IIA, IIB, IIIA, and IIIB diseases, respectively. No patients had stage IIIC disease using the PPS criterion. In the entire cohort, 5260 patients (76.7 per cent) had stage changes and all of them were downstaged (*Table 2*). Among the downstaged patients, 1083 (20.6 per cent) changed by one stage down, 3515 (66.8 per cent) by two stages down, and 662 (12.6 per cent) by three stages down.

**Table 2:** Frequency of stage migration among individual patients

The 7th anatomical staging	The 8th prognostic staging							
	IA	IB	IIA	IIB	IIIA	IIIB	IIIC	Total
IB	300 (100)	0	0	0	0	0	0	300 (4.4)
IIA	807 (74.3)	110 (10.1)	169 (15.6)	0	0	0	0	1086 (15.8)
IIB	62 (2.6)	1727 (72.5)	0	594 (24.9)	0	0	0	2383 (34.7)
IIIA	0	600 (32.1)	790 (42.2)	0	480 (25.7)	0	0	1870 (27.3)
IIIB	0	0	0	0	153 (29.9)	359 (70.1)	0	512 (7.5)
IIIC	0	0	0	0	191 (26.9)	520 (73.1)	0	711 (10.4)
Total	1169 (17.0)	2437 (35.5)	959 (14.0)	594 (8.7)	824 (12.0)	879 (12.8)	0	6862 (100)

Data are presented as *n* (%).

### Survival and staging model fit

**Fig. 2:**
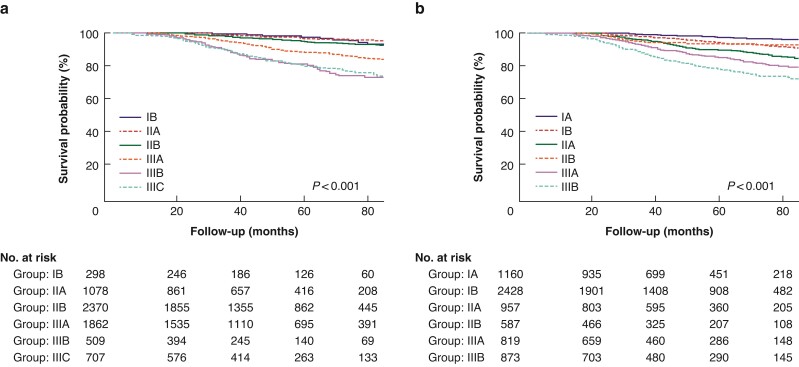
Kaplan–Meier survival curves for breast cancer-specific survival according to the 7th anatomic staging (a) and 8th prognostic staging (b)

With a median follow-up of 48 months (range 0 to 107 months), a total of 648 deaths were observed; 500 died of BC. Overall 5-year BCSS was 90.9 per cent. Using the AS criterion, the 5-year BCSS was 97.6 per cent, 96.8 per cent, 94.9 per cent, 88.5 per cent, 80.4 per cent, and 79.9 per cent in patients with stage IB, IIA, IIB, IIIA, IIIB, and IIIC disease, respectively (*P* < 0.001). However, similar BCSS was found between those with stage IB and IIA diseases (*P* = 0.593), those with stage IIA and IIB disease (*P* = 0.296), and also between stage IIIB and IIIC diseases (*P* = 0.920) (*Fig. 2a*). Regarding PPS, 5-year BCSS was 97.6 per cent, 94.3 per cent, 90.0 per cent, 936 per cent, 85.1 per cent, and 78.2 per cent in patients with stage IA, IB, IIA, IIB, IIIA, and IIIB diseases, respectively (*P* < 0.001) (*Fig. 2b*). However, the BCSS curves between IB and IIB overlapped (*P* = 0.790).

The C-index in PPS was similar to AS (0.690 *versus* 0.682; *P* = 0.346). Further investigating its performance, PPS demonstrated a small difference in AIC (8239 *versus* 8245) compared to AS. Together, the results did not validate a superior predictive value for PPS compared with AS.

### Prognostic analyses

*Table 3* shows the results of Cox regression analyses for BCSS according to AS and PPS. Stage IB and stage IA were used as the reference categories in AS and PPS, respectively. With AS, BCSS of stage IIA (hazard ratio (HR) 0.770, 95 per cent confidence interval (c.i.) 0.359 to 1.650; *P* = 0.501) and IIB (HR 1.361, 95 per cent c.i. 0.681 to 2.680; *P* = 0.390) disease was not statistically different compared with patients with stage IB disease. Regarding PPS, all stage categories showed significantly inferior BCSS compared with patients with stage IA disease.

**Table 3:** Cox regression breast cancer-specific survival analysis

Staging	Anatomical staging*			Prognostic staging†		
	HR	95% c.i.	*P*	HR	95% c.i.	*P*
**IA**	—	—	—	1		
**IB**	1			2.553	1.617–4.031	<0.001
**IIA**	0.770	0.359–1.650	0.501	4.718	2.957–7.529	<0.001
**IIB**	1.361	0.681–2.680	0.390	2.541	1.453–4.444	<0.001
**IIIA**	3.190	1.632–6.236	0.001	6.318	3.974–10.045	<0.001
**IIIB**	5.493	2.748–10.980	<0.001	9.654	6.172–15.101	<0.001
**IIIC**	5.385	2.731–10.620	<0.001	—	—	—

*Adjustment of age, race, histology, tumour grade, and progesterone receptor status.

†Adjustment of age, race, and histology. c.i., confidence interval; HR, hazard ratios.

### The implication of PPS for PMRT decision-making

**Fig. 3:**
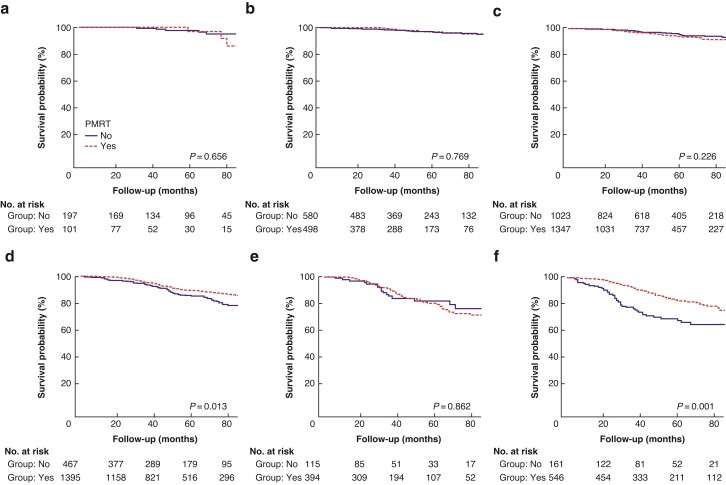
Kaplan–Meier survival curves for breast cancer-specific survival between those with and without postmastectomy radiotherapy (PMRT) according to the 7th anatomic staging

**Fig. 4:**
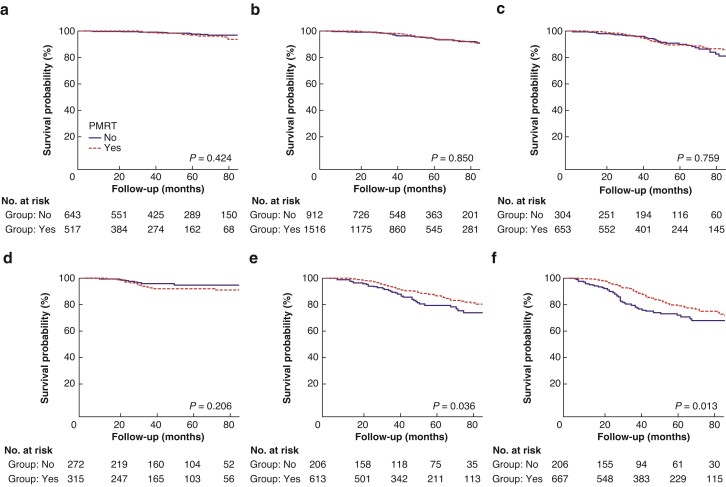
Kaplan–Meier survival curves for breast cancer-specific survival between those with and without postmastectomy radiotherapy (PMRT) according to the 8th prognostic staging

Analyses comparing BCSS for all stage categories using AS and PPS treated with PMRT *versus* no PMRT were conducted (*Table 4*). Regarding AS, patients who received PMRT had significantly better BCSS than those who had not in stage IIIA (HR 0.686, 95 per cent c.i. 0.503 to 0.935; *P* = 0.017) and stage IIIC (HR 0.474, 95 per cent c.i. 0.325 to 0.693; *P* < 0.001) disease. Among patients with stage IB (*P* = 0.675), IIA (*P* = 0.677), IIB (*P* = 0.100), and IIIB (*P* = 0.747) disease, those who received PMRT had similar BCSS to those who had not. The BCSS curves between those with and without PMRT after stratification by AS are shown in *Fig. 3*. When limiting the analyses to patients with PPS, patients who received PMRT had significantly better BCSS than those who had not in stage IIIA (HR 0.640, 95 per cent c.i. 0.420 to 0.976; *P* = 0.038) and stage IIIB (HR 0.653, 95 per cent c.i. 0.460 to 0.926; *P* = 0.017) disease. Among patients with stage IA (*P* = 0.336), IB (*P* = 0.893), IIA (*P* = 0.815), and IIB (*P* = 0.120) disease, those who received PMRT had similar BCSS to those who had not. The BCSS curves between those with and without PMRT after stratification by PPS are shown in *Fig. 4*.

**a** Stage IB disease. **b** Stage IIA disease. **c** Stage IIB disease. **d** Stage IIIA disease. **e** Stage IIIB disease. **f** Stage IIIC disease.

**a** Stage IA disease. **b** Stage IB disease. **c** Stage IIA disease. **d** Stage IIB disease. **e** Stage IIIA disease. **f** Stage IIIB disease.

**Table 4:** Sensitivity analyses of postmastectomy radiotherapy (PMRT) receipt on breast cancer-specific survival using the Cox regression models

Variables	HR	95% c.i.	*P*
**7th AJCC stage IB^a^**			
No PMRT	1		
PMRT	1.355	0.324–5.623	0.675
7th AJCC stage IIA*			
No PMRT	1		
PMRT	0.842	0.375–1.892	0.677
7th AJCC stage IIB*			
No PMRT	1		
PMRT	1.420	0.934–2.157	0.100
7th AJCC stage IIIA*			
No PMRT	1		
PMRT	0.686	0.503–0.935	0.017
7th AJCC stage IIIB*			
No PMRT	1		
PMRT	1.096	0.628–1.913	0.747
7th AJCC stage IIIC*			
No PMRT	1		
PMRT	0.474	0.325–0.693	<0.001
**8th AJCC stage IA†**			
No PMRT	1		
PMRT	1.519	0.648–3.564	0.336
**8th AJCC stage IB†**			
No PMRT	1		
PMRT	1.026	0.704–1.497	0.893
**8th AJCC stage IIA†**			
No PMRT	1		
PMRT	0.948	0.607–1.482	0.815
**8th AJCC stage IIB†**			
No PMRT	1		
PMRT	1.853	0.852–4.032	0.120
**8th AJCC stage IIIA†**			
No PMRT	1		
PMRT	0.640	0.420–0.976	0.038
**8th AJCC stage IIIB†**			
No PMRT	1		
PMRT	0.653	0.460–0.926	0.017

* Adjustment of age, race, histology, tumour grade, and progesterone receptor status. †Adjustment of age, race, and histology. HR, hazard ratio; c.i., confidence interval; AJCC, American Joint Committee on Cancer.

Although this study did not find better BCSS in patients with stage IIIB disease who were treated with PMRT, PMRT should be administrated in patients with stage IIIA, IIIB, and IIIC disease using the AS criterion in the current clinical practice of BC^16^. There were 1870, 512, and 711 patients staged as IIIA, IIIB, and IIIC diseases using the AS criterion (3093 patients in total), respectively. In addition, 824 and 879 patients were reassigned as having stage IIIA and IIIB disease using the PPS criterion (1703 patients in total), respectively, which showed that PPS might allow approximately 1390 patients with stage III disease (45.0 per cent) in the AS criterion to avoid PMRT.

## Discussion

In this study, a population-based cohort was used to assess the performance of the prognostic and predictive value of the 8th AJCC PPS specifically for patients with HoR^+^/HER2^+^ BC with node-positive disease after mastectomy. A similar prognostic performance between AS and PPS was found, despite the finding that PPS reallocated approximately three-quarters of patients to a more favourable stage category and no patients had upstaged. In addition, PPS may also guide the decision-making for PMRT.

Previous large cohort studies have shown a reallocation rate of 46.2 to 54.0 per cent using the PPS system, including 31.1 to 46.2 per cent and 7.5 to 21.2 per cent of patients being downstaged and upstaged, respectively^6,20–23^. However, no studies investigated the discrepancy between AS and PPS for patients with HoR^+^/HER2^+^ BC. Currently, limited studies have reported the discrepancy between AS and PPS specifically for HER2^+^ patients, regardless of HoR status^9,24^. A study from the National Cancer Database (NCDB) showed 29.4 per cent of HER2^+^ patients had stage changes using the PPS criterion and all of them were downstaged^9^, which was similar to the results of Jang *et al.*^24^. In addition, in the results from the ShortHER trial, 41.6 per cent of patients had their stage changed and all were downstaged by PPS (68 per cent of patients were HoR^+^)^14^. The rate of the stage change (76.7 per cent) was higher in this study than in the abovementioned studies, and all cases were downstaged. All patients had ER^+^ disease, which was the main reason for the substantial downstaging in this study.

The current study showed that the substantial downstaging of patients using PPS did not impact the performance of the staging system, which remained similar to AS. This finding was similar to results from the ShortHER trial^14^. In current clinical practice, node-positive HoR^+^/HER2^+^ BC should be treated with chemotherapy, anti-HER2 therapy, and endocrine therapy^16^. Before the anti-HER2 era, patients with HER2^+^ disease had inferior survival outcomes *versus* those with HER2^–^ disease^25,26^, while the prognosis is opposite in the era of anti-HER2 treatment^27,28^. Moreover, the addition of ovarian suppression in premenopausal patients^29^, the use of aromatase inhibitors in postmenopausal patients^30^, and the use of aromatase inhibitors with the addition of ovarian function suppression in premenopausal patients are associated with a better BCSS in HoR^+^ patients^31^. These can partly explain the similar risk stratification between the two staging systems in the current study.

It should be noted that there was no significant difference in BCSS among those with stages IB, IIA, and IIB diseases using the AS criterion. Using the PPS criterion, those with stage IB (HR 2.553) and IIA (HR 4.718) diseases had inferior BCSS compared to stage IA disease. This finding was similar to findings from the ShortHER trial, which reflected contemporary clinical practice for HER2^+^ patients^32^. The PPS has recognized the prognostic effect of biologialc markers in BC, resulting in a substantial downstaging *versus* AS. One of the consequences of staging migration is a better risk stratification for patients with stage I to IIA disease. The main implication of staging migration regarding PPS is more valuable than AS, providing an effective tool for discussing their prognosis with patients. Using the PPS criterion, more patients will be reassigned to a more favourable staging category and will be informed about a better prognosis than the AS.

PMRT should be considered for stage T1-2N1 BC and stage N2/3 BC is an absolute indication^16^. However, owing to the progress of multimodal therapy, the role of PMRT in patients with node-positive BC remains controversial. The findings from the HERA trial showed that PMRT for HER2^+^ patients with N1 disease was associated with a lower LRR rate (*P* = 0.004) and a better disease-free survival (*P* = 0.01) but no improvement in distant metastasis-free survival (*P* = 0.19) or overall survival (OS) (*P* = 0.06)^33^. However, the results from Tseng *et al.* showed a substantially lower LRR rate for HER2^+^ patients who were treated with anti-HER2 therapy (5-year LRR rate 0.26 per cent)^34^. Regarding N2/3 staging, Shi *et al.*, using the data from NCDB, showed that the benefit of PMRT in addition to hormonal therapy alone, chemotherapy alone, or both on OS seems to be marginal and not statistically significant^35^. Although SEER does not record information regarding anti-HER2 treatment, it has been verified that PPS is more valuable in predicting the prognosis than AS in previous validation studies including patients from the SEER database^6–11^. Moreover, the patients included in this study were diagnosed after 2010, and all had received adjuvant chemotherapy; thus, it could be assumed that most patients will have received the corresponding multimodal treatment.

There are no previous studies assessing the role of PMRT in node-positive BC using the PPS criterion. Whether PPS may be of clinical value in identifying potential patients for de-escalated PMRT was explored. Using the AS criterion, patients who received PMRT had significantly better BCSS compared with those who had not in stage IIIA and stage IIIC diseases. Regarding the PPS criterion, patients who received PMRT had significantly better BCSS compared with those who had not in stage IIIA and stage IIIB diseases. PPS might allow approximately 43.5 per cent of stage III patients based on the AS criterion to avoid PMRT. These findings indicate that PPS might also guide treatment de-escalation. The de-escalated strategies based on the PPS ensure the most effective PMRT along with a more rational resource allocation and help oncologists define a reasonable PMRT plan^36,37^.

Several limitations should be emphasized in this study. Firstly, PMRT was not randomized and the radiotherapy fields of PMRT were not specified in the SEER Program. Secondly, systemic treatment options, including chemotherapy regimen, anti-HER2 treatment, and endocrine therapy, were not available in the SEER database. Thirdly, the 8th AJCC PPS includes information on HoR and HER2 status but does not include information regarding lymphovascular invasion, which has also been shown to have additional prognostic value in BC^38^. The Selective Use of Postoperative Radiotherapy After Mastectomy (SUPREMO) trial included patients with stage II (AS) BC receiving adequate contemporary systemic therapy and investigated the effects of PMRT^39^. The results of this trial are expected in 2023. In this trial, lymphovascular invasion was one of the prognostic factors taken into account for risk stratification. The SUPREMO trial is expected to clarify the indications for PMRT in the contemporary era of system therapy. Fourthly, the SEER database also does not include information regarding LRR and distant recurrence, although distant recurrence is the main cause of death for patients with BC. Moreover, the length of follow-up time in this study may still be inadequate for patients with BC because luminal BC relapse late and the benefits of PMRT only manifest after many years. Unfortunately, SEER did not record HER2 status until 2010. Finally, patients aged 70 years or older were excluded in this study because comorbidities were common among older women with BC and those with more comorbidities were less likely to receive PMRT^40^. Despite these limitations, this study was the first, to date, to investigate the effect of the PPS on prognosis and PMRT decision-making in HoR^+^/HER2^+^ BC in the era of modern multimodal therapy.

Although PPS does not provide better risk discriminatory ability in predicting prognosis than AS in patients with node-positive HoR^+^/HER2^+^ BC after mastectomy, PPS is valuable in providing prognostic counseling to patients and may also guide PMRT decision-making. Randomized trials are highly encouraged to determine the role of PPS in PMRT decision-making for this patient subset.

## Data Availability

The data are available in Surveillance, Epidemiology, and End Results programme at https://seer.cancer.gov/, and can be accessed with SEER Research Plus Data, 18 Registries, Nov 2020 Sub (2000-2018).
